# Oncolytic adenovirus H101 ameliorate the efficacy of anti‐PD‐1 monotherapy in colorectal cancer

**DOI:** 10.1002/cam4.4845

**Published:** 2022-06-28

**Authors:** Lili Huang, Huaxin Zhao, Mengying Shan, Hong Chen, Bin Xu, Yang He, Yu Zhao, Zhuqing Liu, Jianhua Chen, Qing Xu

**Affiliations:** ^1^ Department of Oncology, Shanghai Tenth People's Hospital Tongji University School of Medicine Shanghai China; ^2^ Tongji University Cancer Center Shanghai China; ^3^ Anhui Medical University HeFei China; ^4^ Department of Gastrointestinal Surgery Fujian Provincial Hospital Fuzhou China; ^5^ Department of General Surgery, Shanghai Tenth People's Hospital Tongji University School of Medicine Shangai China

**Keywords:** anti‐PD‐1 therapy, CD8^+^ T cells, colorectal cancer, immunotherapy, oncolytic adenovirus

## Abstract

**Background:**

Immune checkpoint blockade therapy with anti‐programmed cell death (PD)‐1 antibodies provides therapeutic effect for many patients of various cancers but remains inadequate in colorectal cancer (CRC) patients. The present study aims to assess the efficacy of oncolytic adenovirus (Onco^Ad^) in enhancing the anti‐PD‐1 treatment of CRC.

**Methods:**

The estimating relative subsets of RNA transcripts algorithm was used for estimating the infiltrated immune cells in melanoma and CRC tissues. The efficacy of Onco^Ad^ with anti‐PD‐1 monotherapy was performed in a CT26 CRC mouse model in vivo. Flow cytometric analysis of peripheral blood and tumor tissues determined the difference anti‐tumor immune efficacy of Onco^Ad^ with anti‐PD‐1 monotherapy.

**Results:**

The Cancer Genome Atlas database indicated that CD8^+^ T cells and regulatory T cells were significantly elevated in melanoma compared to CRC cohorts. Moreover, intratumor injection of oncolytic adenovirus enhanced T cell infiltration and decreased Treg percentages in the CT26 CRC colorectal cancer mouse model. Combinatorial Onco^Ad^ with anti‐PD‐1 antibody treatment markedly enhanced the anti‐tumor efficacy of anti‐PD‐1 by significantly decreasing the tumor volume and reducing tumor growth in a CRC mouse model. To the end, Onco^Ad^ treatment increased the CD8/Treg ratio, indicating that Onco^Ad^ intratumor injection ameliorate the anti‐tumor immune response of anti‐PD‐1 therapy.

**Conclusion:**

The present study elucidates that Onco^Ad^ promotes intratumor T cell infiltration and improves anti‐PD‐1 immunotherapy, thereby providing a potent combinatorial therapeutic strategy for CRC.

## Graphical abstract

Combinatorial treatment with the oncolytic adenovirus and anti‐PD‐1 therapy led to a higher immunotherapeutic efficacy with excellent safety.

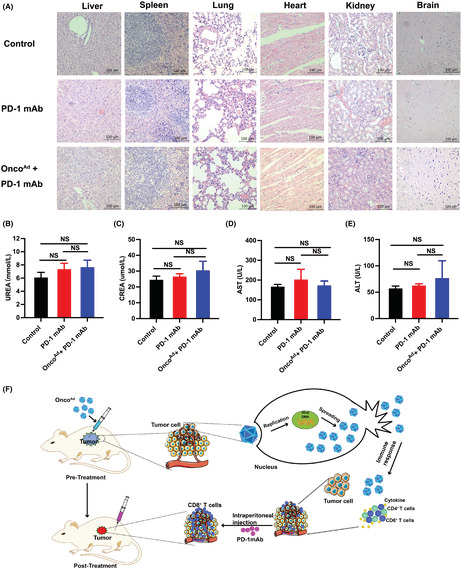

## 1 INTRODUCTION

Tumor immune checkpoint blockade (ICB) therapy to block inhibitory receptors including programmed cell death (PD)‐1/PD ligand (PD‐L) 1, displays excellent efficacy in various cancers.^1^, ^2^ Unfortunately, numerous solid tumors, especial metastatic colorectal cancer (mCRC), have displayed inadequate responses owing to the immunosuppressive tumor microenvironment (TME).^3^, ^4^ The effectiveness of immunotherapy depends on the presence of a baseline immune response and the promotion of pre‐existing immunity.^5^ Based on immune cell infiltration, tumors are categorized as “hot” (high infiltrated) and “cold” (non‐infiltrated).^5^ An effective strategy to overcome the lack of an immune response against a poorly immunogenic tumor is to convert “cold” tumors to “hot” tumors.^6^, ^7^ Thus, combinatorial promising therapy with ICB therapy to enhance T cells response is an emerging therapeutic approach.

Innovative and synergistic combinatorial treatments with ICB therapy are effective strategies for poorly immunogenic tumors.^8^ Oncolytic viruses emerge as a compelling anti‐tumor treatment which can be engineered to selectively replicate within and destroy tumor tissue simultaneously augmenting anti‐tumor immunity.^9^ Previous study reported that intratumor therapy with an oncolytic virus markedly inhibited tumor growth by increasing tumor infiltrated CD4^+^ and CD8^+^ T cells in a melanoma mouse model.^10^ Moreover, Herbst and his colleagues analyzed baseline tumor‐associated immune cells in biopsy specimens of patients treated with anti‐PD‐1 antibodies (PD‐1 mAb) and reported that non‐responders were more likely lacking CD8^+^ T cells within TME.^11^ Increased tumor‐associated CD8^+^ cytotoxic T cells infiltration and elevated PD‐L1 levels enhanced the therapeutic efficacy of PD‐1 mAb.^12^ Thus, combinatorial treatment using oncolytic virus and ICBs can serve as a promising strategy for anti‐tumor therapy.^13^

Colorectal cancer is the third most common cancer and a major cause of cancer‐related mortality worldwide.^14^ When using PD‐1 mAb, clinical mCRC patients with high microsatellite instability (MSI‐H) mutations reportedly responded well to the treatment.^15^ However, only 15% of mCRC patients are with MSI‐H.^16^ It is crucial to develop new practical strategies to enhance ICB efficacy in CRC patients. In a melanoma mouse model, an engineered oncolytic virus that co‐expresses a PD‐L1 inhibitor and GM‐CSF activates tumor neoantigen‐specific T cell responses, increasing the PD‐1/PD‐L1 blockade therapy.^17^ Similarly, oncolytic viruses enhance the sensitivity of ICBs therapy among patients with triple‐negative breast cancer by enhancing the proportion of tumor‐infiltrating immune cells.^18^ Moreover, combinatorial treatment with adenovirus and anti‐PD‐1 therapy inhibits tumor growth, thereby leading to the abscopal effect in a mouse model of lung adenocarcinoma.^19^ However, combinatorial treatment using an oncolytic virus and PD‐1 mAb in CRC treatment is rarely reported. Therefore, Oncolytic viruses can be a potential strategy to improve the response to PD‐1 mAb therapy in CRC.

To develop a new combinatorial treatment, we focused on oncolytic adenovirus H101, a replication‐competent recombinant type 5 human adenovirus engineered by deleting the E1B region, which enhances anti‐tumor immunity upon intratumor injection. This study also describes a combination of oncolytic adenovirus with anti‐PD‐1 increased the anti‐tumor efficiency of ICB therapy in a mouse CRC model, providing a potent combination of therapeutic strategies for CRC therapy.

## 2 MATERIALS AND METHODS

### 2.1 Cell lines and oncolytic adenoviruses

CRC cells (SW620 and CT26), HCC cells (Huh7 and Hepa1‐6), and 293 T cells were purchased from ATCC (Virginia, USA). MC38 cells were obtained from the National Infrastructure of Cell Line Resource (Beijing, China). SW620, MC38, CT26, Huh7, and Hepa1‐6 cells were incubated in Dulbecco's modified eagle medium (Gibco) with 10% fetal bovine serum (Gibco) and 1% penicillin/streptomycin (Gibco).

Oncolytic adenovirus (Onco^Ad^), a recombinant type 5 human adenovirus (H101), was kindly gifted from Shanghai Sunway Biotech Co., Ltd.^20^ The buffer was stored at −20°C and diluted with sterile PBS for further experiments.

### 2.2 Onco^Ad^ replication and cytotoxicity in CRC cells

To assess the cytotoxicity of Onco^Ad^ in tumor cells, CRC cells (SW620, MC38, and CT26) and HCC cells (Huh7 and Hepa1‐6) were seeded in 96‐well wells (5 × 10^3^ cells/well). The next day, Onco^Ad^ suspension was added to the cell culture medium and cells were incubated for 96 h. Cytotoxicity was assessed using the CCK8 assay, and uninfected cells constituted the control group. Viral infection was carried out from 8 to 48 h after Onco^Ad^ incubation, using the adenovirus capsid immunoassay. CRC cells (MC38 and CT26) and HCC cells (Huh7) were incubated into 24‐well plates (2 × 10^5^ cells/well). Onco^Ad^ suspension added to the cell culture medium and the cells were incubated at 37°C with 5% CO_2_ on the following day. At a predetermined timepoint, cells were harvested and fixed with methanol and incubated with anti‐Hexon primary antibody for 1 h at 37°C. Cells were incubated with an HRP‐labeled secondary antibody for 1 h at room temperature, followed by DAB working buffer and incubation for 10 min at room temperature. Positive cells were enumerated microscopically and indicated as the markers of the viral infection.

### 2.3 Mouse model

BALB/c mice were obtained from Shanghai SLAC laboratory animal Co. Ltd. (Shanghai, China). All animal experiments were performed in accordance with the guidelines approved by the institutional Animal Care and Use Committee of Tongji University.

#### 2.3.1 Assessing Onco^Ad^ efficacy in vivo in the CRC mouse model

Six‐week‐old BALB/c mice were included in the study. CT26 cells (5 × 10^5^ cells) were administered into the right limbs of immunocompetent BALB/c mice. Seven days after tumor cell injection (day 0), tumors were measured using the formula (width)^2^ × length/2. Mice were segregated into four groups according to the tumor therapy received: PBS, Onco^Ad^ (5 × 10^8^ VPs of each virus in 100 μl), PD‐1 mAb (10 μg ml^−1^; Bioxcell) or combinatorial treatment with Onco^Ad^ (5 × 10^8^ VPs of each virus in 100 μl) and PD‐1 mAb (10 μg ml^−1^; Bioxcell). On day 0, Onco^Ad^ or PBS were directly injected into the tumor. A second dose (PBS, Onco^Ad^ [5 × 10^8^ VPs of each virus in 100 μl]) was administered on day 4. Anti‐PD‐1 therapy developed herein was administered on days 8, 10, and 12 intraperitoneally, and a replicate for each treatment group for each day. On day 14, all mice were euthanized and organs were harvested for flow cytometry and histopathological analysis.

#### 2.3.2 Flow cytometric analysis of peripheral blood

After treatment, mouse blood samples were obtained and treated with RBC lysis buffer. PBMCs were cultured in RPMI 1640 medium and then probed with different antibodies at 4°C for extracellular and intercellular staining. Fixable viability Dye eFluor™ 780 (Invitrogen) was used to exclude dead cells from subsequent flow cytometric analysis. For extracellular staining, single cells were incubated with anti‐mouse BUV395‐CD45, PE‐Cy7‐CD4, BB700‐CD8, BV480‐CD25, BV605‐PD‐1, BV421‐PD‐L1, PE‐Tim‐3 with staining buffer at 4°C in the dark for 30 min. Thereafter, intracellular staining (Alexa Fluor 647‐Foxp3) was performed after samples were fixed and permeabilized with a fix‐perm buffer for 45 min. Various antibodies and other reagents were obtained from BD Bioscience. All buffer solutions were used in accordance with the manufacturer's instructions (BD Bioscience). The CD4^+^ T cells, cytotoxic CD8^+^ T cells, and regulator T cells (Tregs) were analyzed via flow cytometry.

#### 2.3.3 Immunotype analysis of tumors

On day 14, tumors were harvested and cut into small pieces. After tumor pieces were minced and pestled, suspensions were passed through a 70‐μm cell strainer (Corning). Single‐cell suspensions were obtained through lysis with RBC lysis buffer. Tumor‐infiltrating immune cells were analyzed using flow cytometry to assess immunophenotypes. Proportions of tissue‐associated CD4^+^ T cells, CD8^+^ T cells, and Tregs were assessed through flow cytometry. Furthermore, the immune functions of checkpoint receptors were assessed in the tumor.

#### 2.3.4 Histopathological and immunohistochemical staining

Tumor associated immune cells were performed and analyzed using CRC and paired healthy tissues with histopathological and immunohistochemical staining (IHC). Anti‐CD4, anti‐CD8, anti‐CD206, and anti‐Foxp3 antibodies were used for IHC. Images were obtained using NanoZoomer S210 (Hamamatsu, Hamamatsu, Japan) with a 10× objective. Quantification of positive cells were analyzed using ImageJ software (National Institutes of Health, Bethesda, MD, USA). Moreover, mouse tumor tissues and some organs were harvested and fixed with paraformaldehyde and embedded in paraffin. The samples were cut into 5‐μm‐thick sections for hematoxylin and eosin (H&E) staining. Histopathological toxicity was assessed in the main organs, including the liver, heart, kidneys, and lungs. Tumor H&E and TdT‐mediated dUTP Nick‐End Labeling (TUNEL) staining were performed to determine the different treatments' efficacies. Immunohistochemical staining including CD45, CD4, and CD8 were performed for all CRC tissues. Digitally scanned slices were imaged using a 5× and 20× objective lens. Statistical differences were calculated by ImageJ software after four treatments.

### 2.4 The Cancer Genome Atlas (TCGA) analysis

Cell‐type identification by estimating relative subsets of RNA transcripts (CIBERSORTx) algorithm was used to determine and analyze immune cell infiltration in melanoma and CRC tissues. Twenty‐two immune cell subtypes were identified and analyzed from the annotated gene signature LM22 and 100 permutations of CIBERSORTx web portal (http://cibersortx.stanford.edu/). All samples were enumerated and analyzed in accordance with the CIBERSORTx *P*‐value and root‐mean‐square error. Wilcoxon's test was performed to examine differences in immune absolute score between melanoma and CRC tissues. The CRC (*n* = 343) and skin cutaneous melanoma datasets (SKCM) (*n* = 76) were downloaded from TCGA database using Tumor Immune Estimation Resource (TIMER2.0) (http://timer.cistrome.org). Patients identified from TCGA database were segregated into two quartiles in accordance with the levels of intratumor immune cell infiltration (CD8^+^ Tcells, CD4^+^ T cells, Tregs, and M2 macrophages). Survival rates were analyzed in the high‐ and low‐infiltration groups. The actual numbers of patients involved were also determined by TCGA database.

### 2.5 Biochemical or hematological data

Six‐week‐old BALB/c mice with tumors were included in the study. Mice were segregated into four groups according to the tumor therapy received: PBS, Onco^Ad^, PD‐1 mAb, or combinatorial treatment with Onco^Ad^ and PD‐1 mAb. On a determined day, blood was collected and analyzed for hematological and biochemical (AST, ALT, CREA, and UREA) analysis. Mouse hematological analysis were performed by MINDRAY animal automatic blood cell analyzer (BC‐2800vet, Guangzhou, China). Liver and kidney biochemical analyses were determined by AST, ALT, CREA, and UREA ELISA kit (Rayto, Guangzhou, China).

### 2.6 Statistical analysis

Data were analyzed using GraphPad Prism v.8.0 software (San Diego, CA, USA). A two‐tailed paired or unpaired Student's *t*‐test was performed to analyze differences between groups. One‐ or two‐way ANOVA with multiple comparison correction was performed for multiple‐group comparisons. Statistical significance is indicated as *p < 0.05, **p < 0.01, ***p < 0.001, ****p < 0.0001.

## 3 RESULTS

### 3.1 Poor immunogenic state of CRC tissue

**FIGURE 1:**
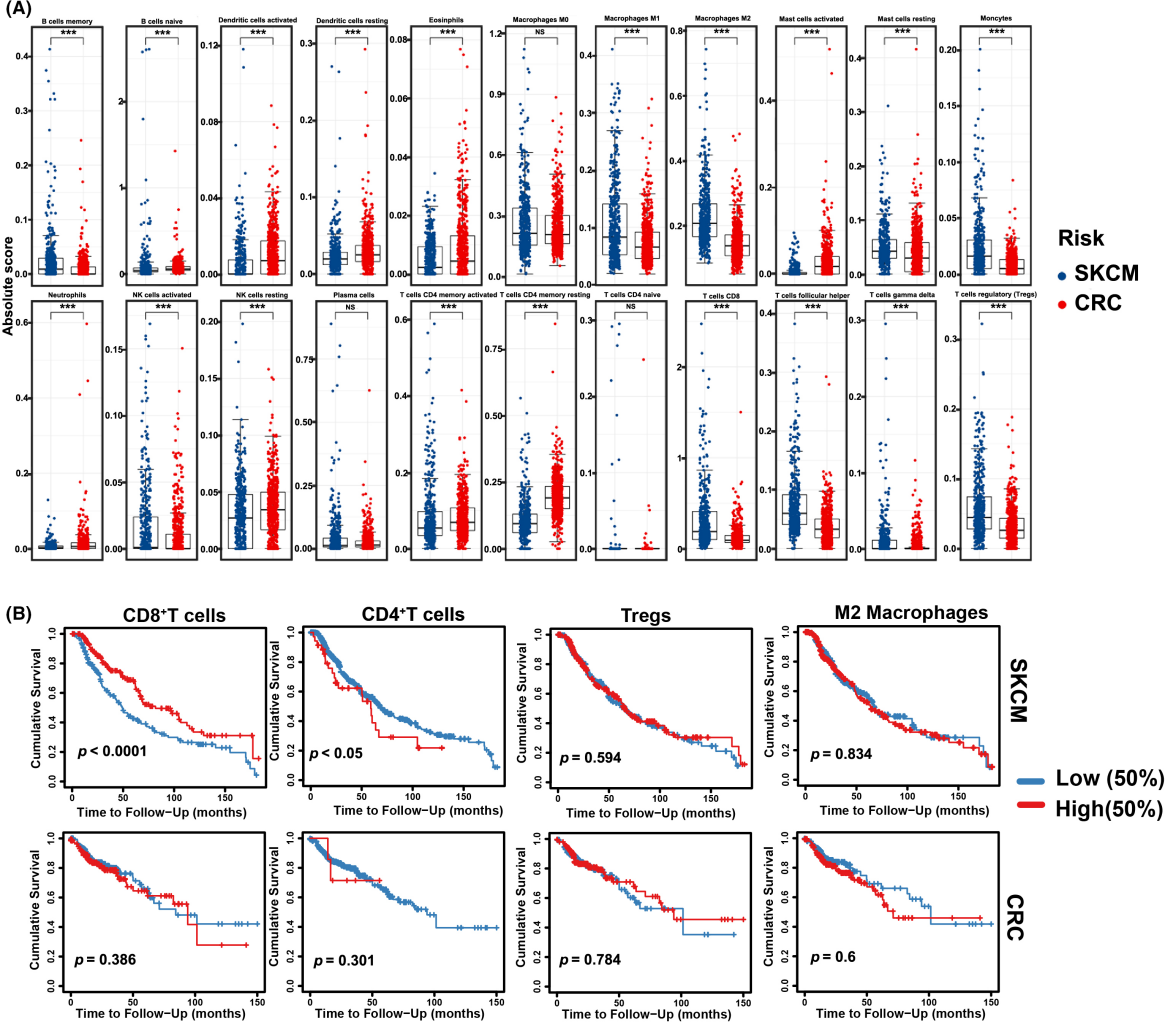
Immune states of colorectal cancer and skin cutaneous melanoma. (A) Boxplots of different immune cells between skin cutaneous melanoma patients (SKCM, *n* = 343) and human colorectal cancer (CRC, *n* = 76) from TCGA dataset using the CIBERSORTx algorithm. The blue boxplot (NC) represents SKCM; the red boxplot (T) represents CRC. (B) Survival curve of CD8^+^, CD4^+^, Tregs, and M2 macrophages in CRC and SKCM. Significance is indicated as *p < 0.05 or **p < 0.01 or ***p < 0.001.

We first determined the tumor infiltrated immune cells in CRC using TCGA database. Twenty‐two immune cell subtypes and immune score were analyzed from human CRC tissues (*n* = 343) and compared to melanoma tissues (*n* = 76). We observed 22 immune cells with different levels in the microenvironment of both melanoma and CRC (Figure 1A). In the adaptive immune response in CRC, the frequencies of CD8^+^ T cells were lower than those in melanoma tissue, probably because CRC is a poorly immunogenic tumor (Figure 1A). Interestingly, tumor associated Treg cells presented ~0.02 score and ~0.05 score in CRC and tumors. In both tumors, M0 macrophages often present higher scores in immune cells, but their scores were only slightly decreased in CRC (NS) compared to melanoma (Figure 1A). We conclude that CRC tumor tissue emerges as a poorly immunogenic tumor with low immune scores.

Given the critical role of T cells in CRC tissues, we next analyzed the immune cell infiltration in human CRC tissues and paired normal tissues. We found that CRC infiltrated CD4, CD8, CD206, and Foxp3 cells were increased when compared with normal tissues, indicating that immune cell infiltration was of high clinical importance in the development of CRC (Figure S1A–E). Based on the tumor infiltrated immune cells, we examined the correlation between immune cells and overall survival (OS) of CRC patients. Roles of immune cells were investigated using CRC TCGA database (Figure 1B). As expected, there is no significant survival outcomes of immune cell infiltration in CRC cohorts (Figure 1B). We observed that survival outcomes were significantly higher in melanoma with CD8^+^ T cells infiltration, implying a better patient prognosis concurrent with a previous report^21^ (Figure 1B). Therefore, CRC manifests a poor immunogenic state even in the presence of immune infiltrated.

### 3.2 Low cytotoxicity of Onco^Ad^ on CRC cells in vitro

**FIGURE 2:**
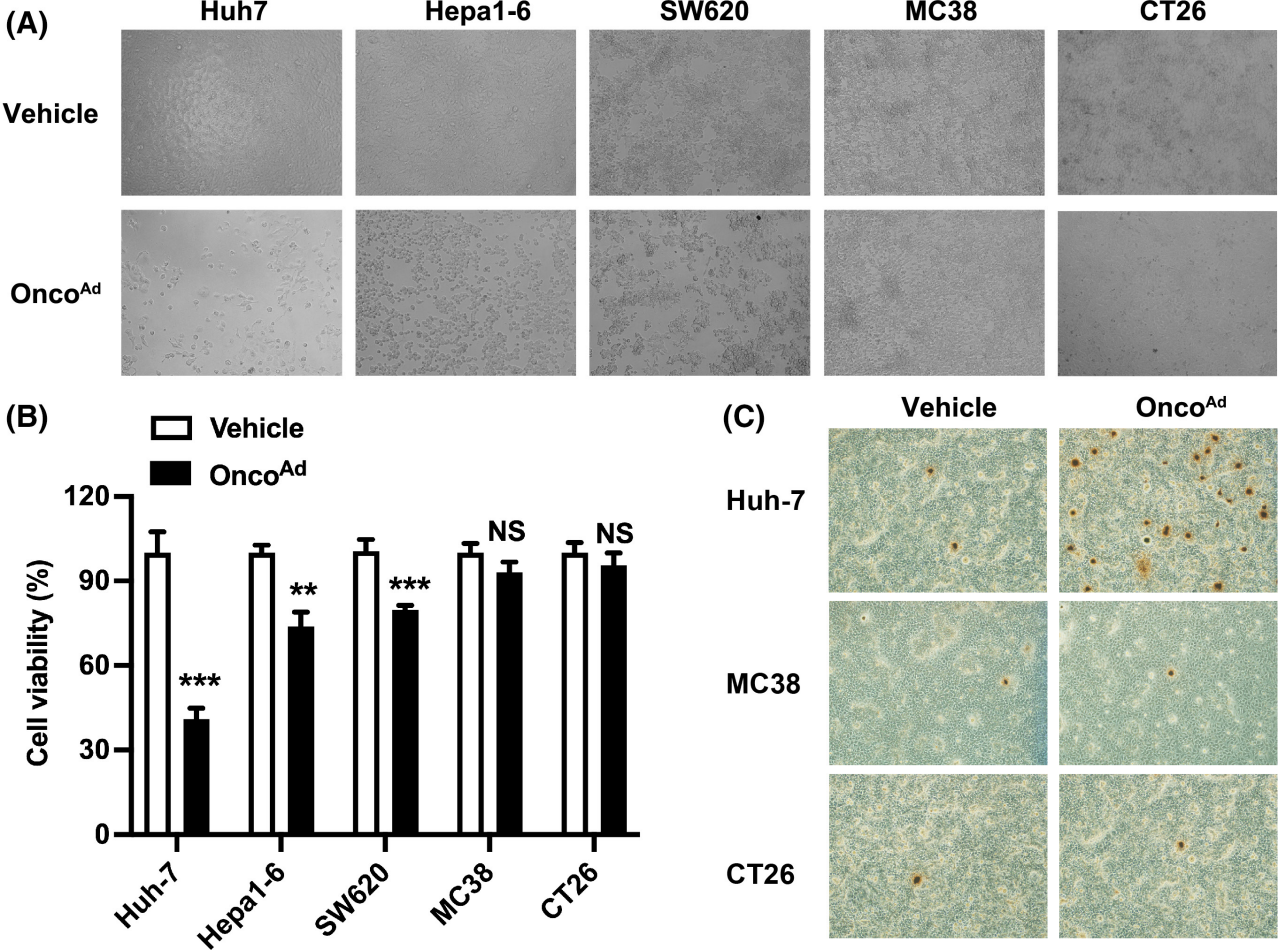
Characteristics of Onco^Ad^ in vitro. (A) Cytotoxicity of Onco^Ad^ in CRC cells (SW620, MC38, and CT26) and HCC (Huh7 and Hepa1‐6). Representative images displayed cell viability after 48 h of Onco^Ad^ treatment. (B) Quantification of positive tumor cells. Significance was determined using the *t*‐test, and all data are presented as mean ± SEM values. Significance is indicated as *p < 0.05 or **p < 0.01 or ***p < 0.001. (C) Proliferation of Huh7, and CT26 cells after Onco^Ad^ infection. Brown represents positive infected cells.

Given the crucial role of Onco^Ad^ in promoting anti‐tumor immunity, we next analyzed the impact role of Onco^Ad^ in ICB therapy on CRC tumors. We first determined the characteristics of Onco^Ad^ by assessing their replication and cytotoxicity in human (SW620 and Huh7) and mouse cancer cells (MC38 and Hepa1‐6) in vitro. The cytotoxicity of Onco^Ad^ and PBS were compared in human and mouse CRC and hepatocellular carcinoma (HCC) cells. Onco^Ad^ displayed significant high cytotoxicity in human CRC and HCC cells and mouse Hepa1‐6, suggesting that Onco^Ad^ present cytotoxicity in cancer cells in vitro (Figure 2A,B). However, MC38 and CT26 cells showed no statistically different in Onco^Ad^ treatment compared to the control (Figure 2A,B). Moreover, Onco^Ad^ replicated rapidly in human HCC cells (Huh‐7) but not in mouse CRC cells (MC38 and CT26) (Figure 2C). Previous studies showed that Onco^Ad^ could prevent tumor growth in the mouse model of CRC.^22^ The contradictory results of Onco^Ad^ both in vitro and in vivo suggested that there might be the presence of additional mechanisms of action of Onco^Ad^ in CRC in vivo.

### 3.3 Onco^Ad^ increased anti‐tumor immunity in CRC in vivo

**FIGURE 3:**
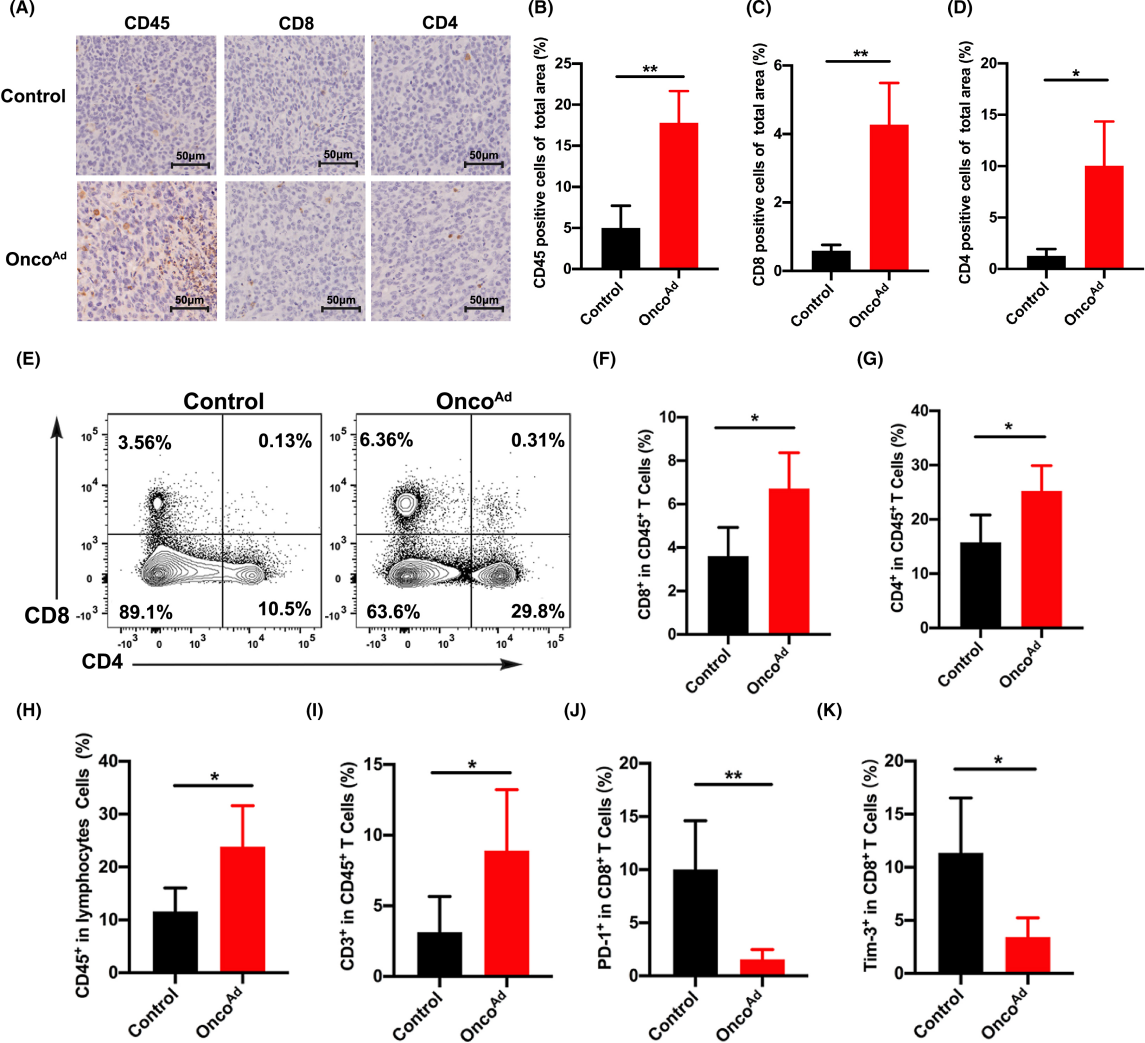
Anti‐tumor response of Onco^Ad^ in the CT26 mouse model. (A) Immunohistochemical staining for CD45, CD4, and CD8 in the tumor after Onco^Ad^ therapy. (B–D) Quantification of positive cells via ImageJ software following CD45 (B), CD8 (C), and CD4 (D) staining (*n* = 3, *p < 0.05 and **p < 0.01). (E–I) The proportion of tumor‐infiltrating immune cells treated with intratumor Onco^Ad^. Representative contour plots of CD4^+^ and CD8^+^ T cells (E) in the CRC mouse model (left); the percentages of positive CD4^+^ and CD8^+^ T cells (F, G) are indicated on the right. Analysis of the phenotypes of CD45^+^ T cells (H) and CD3^+^ T cells (I) after Onco^Ad^ therapy. (J, K) Representative contour plots of PD‐1 (J) and Tim‐3 expression (K) of CD8^+^ T cells in the CRC mouse model. Significance is indicated as *p < 0.05 or **p < 0.01.

We next established a CT26 mouse model to explore the underlying mechanism of Onco^Ad^ therapy in CRC. Immunohistochemical staining was performed to confirm the therapeutic efficacy of Onco^Ad^ in CRC mouse model. We found that Onco^Ad^ treatment elucidated higher tumor cell necrosis and apoptosis in CRC than control groups, implying excellent therapeutic efficacy (Figure 3A). To explore the differences of anti‐tumor immunity, we performed CD45, CD4, and CD8 staining in mouse tumor tissues (Figure 3A). Marked differences were observed in tumor‐infiltrated immune cells (TILs) between the Onco^Ad^‐treated and PBS‐treated groups (Figure 3A–D). As expected, the proportion of CD45^+^ TILs were higher in the Onco^Ad^‐treated group than in the control group (Figure 3A,B). We evaluated the distribution of CD4^+^ T cells and CD8^+^ T cells in tumor tissues, which was considered to reflect adaptive immune response during cancer therapy. Our results showed that the proportion of CD8^+^ T cells was higher in the Onco^Ad^‐treated group than in the PBS‐treated group (Figure 3C). Similar trends were observed for CD4^+^ T cell infiltration (Figure 3D). Moreover, we analyzed the tumor‐infiltrating lymphocytes in mice using flow cytometry (Figure 3E). The percentages of tumor‐infiltrating immune cells including CD8^+^ T cells and CD4^+^ T cells were increased upon Onco^Ad^ treatment (Figure 3E–G). Similarly, the proportion of CD45^+^ T cells and the CD3^+^ T cells were increased following intratumor Onco^Ad^ treatment in CRC (Figure 3H,I). The flow cytometry data were concurrent with those of IHC of tumor tissue. Collectively, these results indicate that Onco^Ad^ therapy improves the anti‐tumor immune response in CRC. Analysis of the functional state of immune cells, evaluated on the basis of the expression levels of checkpoint receptors, revealed that Onco^Ad^ treatment decreased the proportion of PD‐1 expression on tumor infiltrated CD8^+^T cells (Figure 3J). Furthermore, Onco^Ad^ treatment downregulated Tim‐3 expression on the tumor‐associated CD8^+^T cells (Figure 3K). Therefore, immune checkpoint molecules on the CD8^+^T cells were decreased in the intratumor Onco^Ad^‐treated group compared to PBS‐treated group, indicating an enhanced immunotherapeutic efficacy in CRC. Taken together, these results indicate that Onco^Ad^ therapy not only inhibits tumor growth but also promotes anti‐tumor immunity.

Onco^Ad^ treatment has been previously administered during immunotherapy for numerous cancers. We collected blood samples from CT26 mice to evaluate the immune response to intratumor Onco^Ad^ administration. As shown in Figure S2A–C, intratumor injection of Onco^Ad^ treatment decreased the proportion of CD4^+^ and CD8^+^ T cells in the blood samples; however, the percentages of CD45^+^ T cells remained unchanged. Moreover, Onco^Ad^ therapy significantly decreased the proportion of CD4^+^CD25^+^Foxp3^+^Treg cells among CD45^+^ T lymphocytes (Figure S2D). Consequently, the CD8^+^ T cells/Treg ratio increased after intratumor Onco^Ad^ treatment in CRC compared to control ones (Figure S2E). Therefore, Onco^Ad^ intratumor therapy improves systemic immunity in CRC, providing a potential treatment strategy for CRC.

### 3.4 Intratumor Onco^Ad^ injection enhanced the anti‐tumor efficacy of anti‐PD‐1 in mice with CRC

**FIGURE 4:**
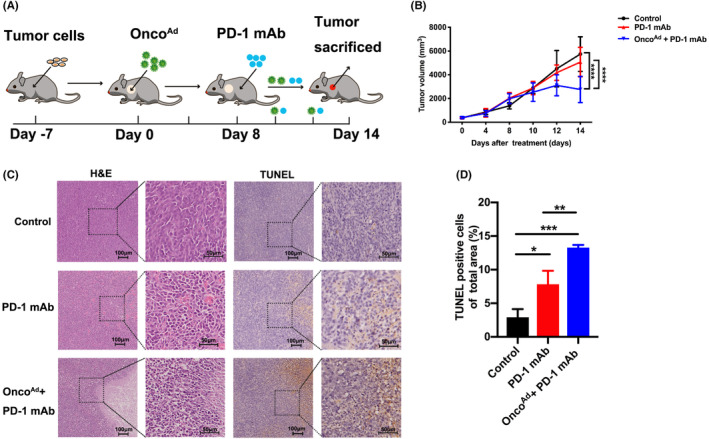
Anti‐tumor efficacy of combinatorial treatment with Onco^Ad^ and PD‐1 mAb therapy in the CT26 mouse model. (A) Schematic representation of different treatments given in the present study. (B) Relative tumor volume curve of CT26 tumors after treatment with PD‐1 mAb, and combinatorial Onco^Ad^ and PD‐1 mAb therapy (*n* = 5; *p* < 0.05). (C) Tumor necrosis in tumor sections, as indicated through H&E staining. Apoptosis in tumor sections was examined through TUNEL staining. (D) Quantification of positive cells via ImageJ software following TUNEL staining (*n* = 5; *p* < 0.01). Scale bar = 50 or 100 μm. Significance is indicated as *p < 0.05 or **p < 0.01.

Finally, we analyzed the impact of Onco^Ad^ combined ICB on tumor growth in CRC (Figure 4A). All treatments inhibited tumor growth, but the combined therapy had a stronger inhibitory effect compared with the monotherapy (Figure 4B). H&E and TUNEL staining confirmed the combinatorial effect with Onco^Ad^ and PD‐1 mAb in mouse cancer tissues (Figure 4C,D). We found that combined Onco^Ad^ and PD‐1 mAb treatment increased the tumor cell necrosis and apoptosis in CRC when compared to the anti‐PD‐1 group, implying excellent therapeutic efficacy (Figure 4C,D). These results indicate that combinatorial Onco^Ad^ and PD‐1 mAb therapy is an excellent therapeutic option that leads to promising anti‐cancer effects in mice with CRC.

**FIGURE 5:**
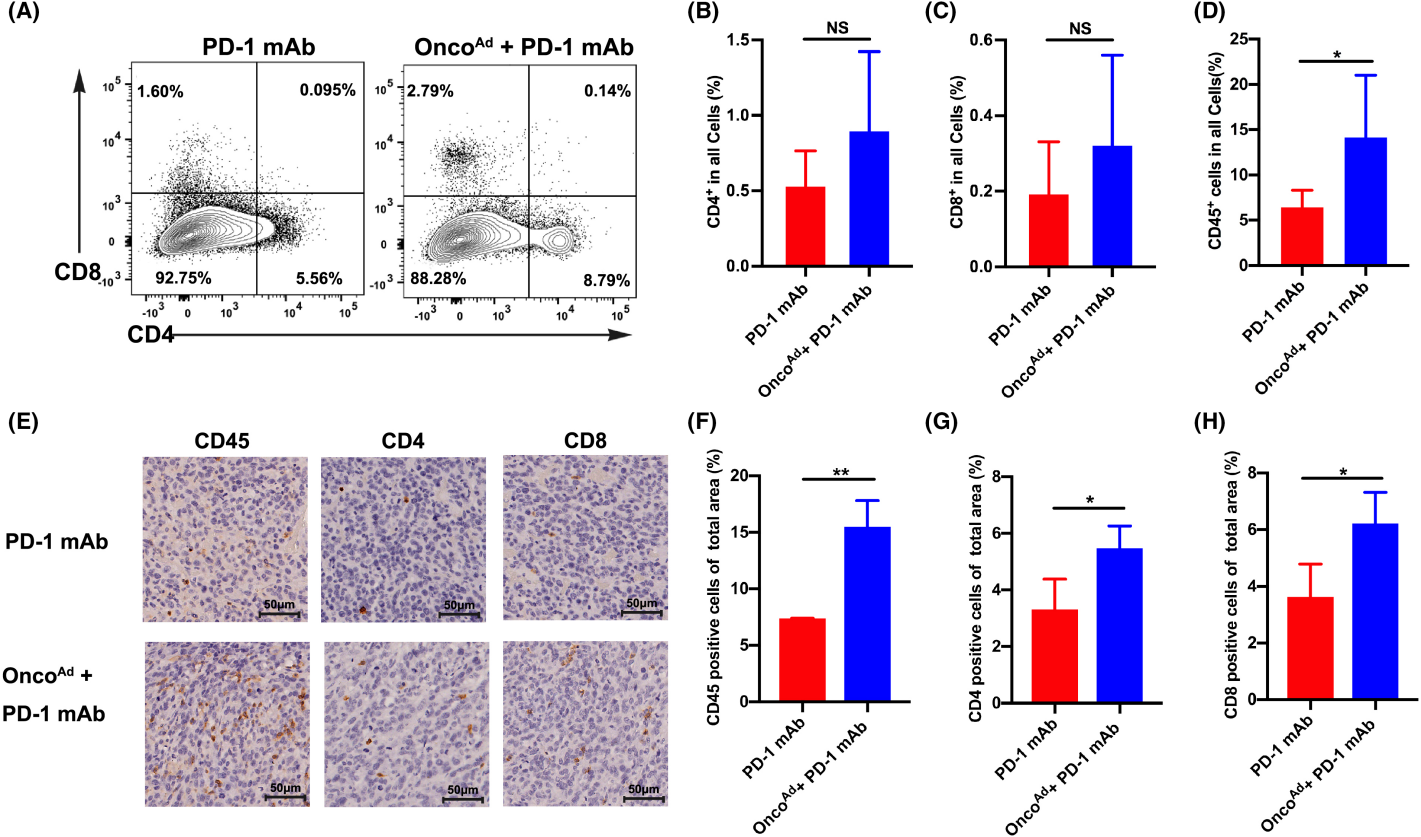
Onco^Ad^ enhanced the immune response of PD‐1 mAb therapy in the CT26 mouse model. (A–C) Representative contour plots of CD4^+^ and CD8^+^ T cells in the CRC mouse model (left) after combinatorial Onco^Ad^ and PD‐1 mAb therapy; percentages of CD4^+^ and CD8^+^ T cells are indicated on the right (B, C). (D) Percentages of immune cells upon combinatorial Onco^Ad^ and PD‐1 mAb therapy compared to PBS treatment. Analysis of the phenotype of CD45^+^ T cells (D) after combinatorial Onco^Ad^ and PD‐1 mAb therapy. (E) Immunohistochemical staining for CD45, CD4, and CD8 in the tumor after combinatorial Onco^Ad^ and PD‐1 mAb therapy. (F–H) Quantification of positive cells via ImageJ software following CD45 (F), CD4 (G), and CD8 (H) staining. Significance is indicated as *p < 0.05 or **p < 0.01.

Onco^Ad^ treatment markedly increased CD8^+^ T cell infiltration and decreased the tumor‐associated Treg proportion in CRC. We analyzed the immune cells to determine the effect of combinatorial Onco^Ad^ and ICI therapy on anti‐tumor immunity. Next, we investigated the role of CD8^+^T cells and Treg cells in Onco^Ad^ and PD‐1 mAb treated tumors. The frequency of CD8^+^ and CD4^+^ T cells slightly increased after the combinatorial Onco^Ad^ and ICB therapy (Figure 5A–C). Compared to anti‐PD‐1 therapy alone, combinatorial therapy markedly increased the proportion of CD45^+^ T cells (Figure 5D), suggesting a higher extent of immune cell infiltration. To confirm the beneficial effects of combinatorial Onco^Ad^ and anti‐PD‐1 therapy, we performed IHC using mouse tumor tissues. As expected, the tumor infiltrated CD45^+^ cells were higher in the combinatorial Onco^Ad^ and anti‐PD‐1 group than in the control group (Figure 5E,F). Moreover, the distribution of CD4^+^ T cells and CD8^+^ T cells in tumor tissues was also checked to assess the immune response after combinatorial Onco^Ad^ and PD‐1 mAb therapy (Figure 5G,H). Similar to the flow results, combinatorial therapy improved the proportion of CD4^+^ T cells in TME when compared with anti‐PD‐1 therapy (Figure 5G). And we found that CD8^+^ T cell infiltration was increased in the combinatorial Onco^Ad^ and PD‐1 mAb group than in the PD‐1 mAb‐treated group (Figure 5H). Thus, we concluded that Onco^Ad^ induced changes (increased the proportion of CD8^+^T cells) promoting the anti‐tumor immunity of PD‐1 mAb in the TME.

**FIGURE 6:**
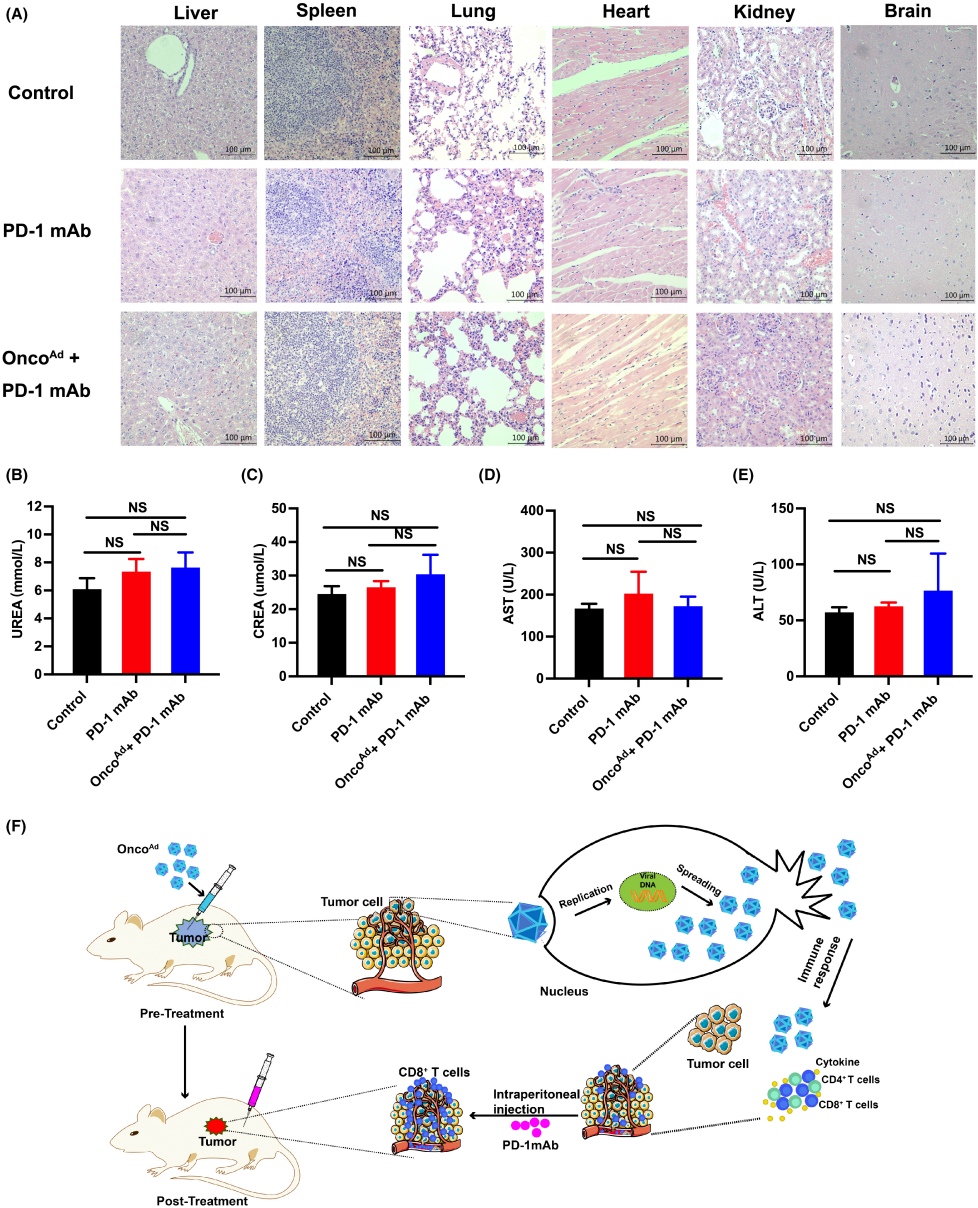
Immune response of tumor microenvironment after combinatorial Onco^Ad^ and PD‐1 mAb therapy in the CT26 mouse model. (A) Organ toxicity was assessed through H&E staining of samples of the liver, spleen, lung, hearts, kidneys, and brain (*n* = 3, *p < 0.05 and **p < 0.01). Magnification 10×. (B–E) Biochemical analysis after combinatorial treatment with Onco^Ad^ and PD‐1 mAb in CRC mouse model. Quantification ALT, AST, CREA, and UREA are presented. (F) Schematic mechanism of combinatorial treatment with Onco^Ad^ and PD‐1 mAb therapy in the CRC mouse model.

Since combined ICB therapy presented far more toxic than monotherapy, systemic toxicity was trafficked in the therapeutic process. We found that combinatorial Onco^Ad^ and PD‐1 mAb therapy did not damage organs including the liver, kidneys, heart, and lungs (Figure 6A). Besides, biochemical results showed that Onco^Ad^ treatments did not exert cytotoxic effects in specific organs (liver, kidneys, and bone marrow) (Figure 6B–E). These data demonstrated that Onco^Ad^ therapy did not damage metabolic organs in the CRC mouse model. In the CRC mouse model, intratumor injection of Onco^Ad^ mediating growth and immunity correlates closely with the efficacy of treatment (Figure 6F). Taken together, these data show that Onco^Ad^ treatment enhances the therapeutic efficacy of PD‐1 mAb therapy for CRC treatment.

## 4 DISCUSSION

The anti‐tumor response of the oncolytic viruses has been investigated in preclinical models and clinical trials.^23^ Oncolytic viruses can target and eliminate tumor cells, activate the immune system, and enhance the anti‐tumor response.^19^ However, combinatorial treatment using an oncolytic virus and PD‐1 mAb in CRC treatment is rarely reported. Therefore, Onco^Ad^ treatment can be a potential strategy for improving the response to anti‐PD‐1 therapy in CRC. In this study, a recombinant human oncolytic virus (H101) was selected as a therapeutic agent for CRC. Intratumor injection of the oncolytic virus induced the recruitment of CD8^+^ T cells from peripheral tissues to TME and decreased the proportions of tumor‐infiltrating Tregs. We investigated the efficacy of combinatorial treatment with Onco^Ad^ and immune checkpoint receptor inhibitors anti‐PD‐1 in a CRC mouse model. Our results provide evidence that combinatorial Onco^Ad^ and ICB therapy significantly enhanced the anti‐tumor efficacy of anti‐PD‐1 by increasing CD8^+^ T cell infiltration and reducing the tumor volume in CRC.

Notably, Onco^Ad^ display significant tissue tropism among patients with malignant cancers without severe side effects.^24^ Adenoviral replication is initiated by the E1 region, which encodes transcriptional units E1B, E2, and E3.^25^ Interestingly, Onco^Ad^ selectively replicates in p‐53 mutated neoplasms, leaving normal cells unaffected.^25^ Therefore, oncolytic adenoviruses treatment can lead to marked cytotoxicity in cancer cells and facilitate a safe therapeutic strategy. Our previous work has demonstrated that there was no replication of recombinant human type‐5 adenovirus in mouse live cancer cells.^26^ Similarly, Onco^Ad^ displayed no significant cytotoxicity for CRC cells in vitro in this study. When compared to human cell lines, infection mouse BALB/c 3 T3 cells by adenovirus 5 resulted in at least 1000‐fold lowered yields due to limited viral gene expression level.^27^ Steady levels of DNA and RNA were significantly decreased in the infected mouse cells, and the early region 1A(E1A) and E1B mRNAs were reduced, which their proteins were hardly detectable in vitro.^28^ While, Onco^Ad^ therapy displayed a high anti‐tumor response both in mouse liver cancer and in CRC mouse model in the present study. The contradictory results of Onco^Ad^ both in vitro and in vivo suggested that there might be the presence of additional mechanisms of action of Onco^Ad^ in promoting therapeutic effect of CRC in vivo. Thus, more knowledge about the underlying mechanisms of Onco^Ad^ therapy is paramount importance in CRC. And a preclinical study on Onco^Ad^ treatment would be a crucial step to generate a potential application in the combinatorial treatment of CRC patients.

Several mechanisms are involved in the anti‐tumor effects of oncolytic viruses' treatment, including the regulation of gene expression and cancer cell metabolism, and tumor immune status.^29^ Recent studies demonstrated that oncolytic adenoviruses could upregulate TNF‐α production, resulting in cancer cell apoptosis and necrosis.^30^ In the B16‐OVA syngeneic mouse model and ovarian cancer, oncolytic adenoviruses increased the proportion of tumor‐infiltrating CD8^+^ T cells and CD4^+^ T cells, leading to significant reduction in tumor growth.^31^, ^32^ Herein, we used oncolytic adenovirus to explore the underlying mechanisms in CRC using a BALB/c mouse model. Flow cytometric data revealed that Onco^Ad^ treatment decreased the proportion of CD4^+^CD25^+^Foxp3^+^Treg cells and increased the CD8/Treg ratio in peripheral blood, suggesting that systematical immunity enhanced after Onco^Ad^ treatment. Furthermore, an increase in the number of tumor‐infiltrating CD8^+^ T cells and a reduction of Tregs were also observed in the Onco^Ad^ treatment group compared to the PBS‐treated group. Besides, immune checkpoint receptors on CD8^+^ T cells were downregulated upon intratumor Onco^Ad^ injection in CRC tissue. Based on these results, we concluded that oncolytic adenoviruses treatment presented excellent therapeutic effect by increasing the anti‐tumor immunity including tumor infiltrated CD8^+^T cells. Reports demonstrated that non‐anti‐PD‐1 responders were more likely to lack CD8^+^ T cells in TME.^11^ Increased infiltration of tumor‐associated CD8^+^ cytotoxic T cells can enhance the therapeutic efficacy.^12^ According to the above data, we hypothesize that Onco^Ad^ intratumoral treatment could improve the efficacy of anti‐PD‐1 therapy in CRC patients.

Although oncolytic adenoviruses displayed an excellent anti‐tumor response, the therapeutic potential of Onco^Ad^ was limited by the host immune response in vivo. Thus, combinatorial treatment with other anti‐cancer therapies should be investigated to prolong the efficacy of the oncolytic virus. Patients receiving ONCOS‐102 treatment displayed PD‐L1 upregulation in melanoma cells,^33^ and the results showed that combinatorial ONCOS‐102 and anti‐PD‐L1 emerged an enhanced anti‐tumor efficacy.^34^ In patients with multiple myeloma, oncolytic virotherapy emerge as an antigen agnostic vaccine by increasing cytotoxic T cell response, providing a potential treatment in combination with ICB.^35^ Recent studies demonstrated that oncolytic adenovirus inhibited tumor growth in CRC by suppressing cell proliferation, metastasis, and tumor stemness.^36^ As the limitation of anti‐PD‐1 therapy in clinical CRC, it is essential to explore practical strategies to enhance the responsiveness to ICB therapy among CRC patients.^3^ In our work, combinatorial treatment with oncolytic adenovirus and anti‐PD‐1 monotherapy markedly reduced the tumor growth and increased the tumor associated CD8/Treg ratio, promoting the ICB sensitivity in a CRC mouse model. Due to animal ethics and time limit, the therapeutic efficacy is not significant in the oncolytic adenovirus and anti‐PD‐1 treatment. Our current results highlight that the oncolytic virus in combination with other therapeutic modalities, especially with anti‐PD‐1 therapy, offers renewed hope for effective treatment of patients with CRC.

This study shows that treatment with an oncolytic adenovirus effectively prevents tumor growth of CRC in a mouse model. Herein, combinatorial treatment with the oncolytic adenovirus and PD‐1 mAb therapy revealed a high immunotherapeutic efficacy with excellent safety; however, elucidation of underlying mechanisms warrants further research in this field. Moreover, studies should be designed to investigate the potential synergism of the oncolytic adenovirus with other ICBs or immune agents. In summary, combinatorial treatment with an oncolytic adenovirus and PD‐1 mAb therapy improves the anti‐tumor response in CRC, thereby offering a promising strategy to treat patients with CRC.

## AUTHORS CONTRIBUTION

Lili Huang: Conceptualization, Software, Writing‐Original draft preparation; Huaxin Zhao: Methodology, Data curation; Mengying Shan: Data curation; Hong Chen: Data curation; Bin Xu: Data curation; Yang He: Methodology; Yu Zhao: Software; Zhuqing Liu: Visualization, Supervision; Jianhua Chen: Writing‐Reviewing and Editing; Qing Xu: Supervision, Validation.

## CONFLICT OF INTEREST

The authors have no conflicts of interest to report for this article. The authors certify that they have no affiliations with or involvement in any organization or entity with any financial interest in the subject matter or materials discussed in this manuscript.

## ETHICS STATEMENT

As all data were publicly available, no ethics approval was sought.

## Supporting information


Figure S1
Figure S2

## Data Availability

The data that support the findings of this study are available from the corresponding author upon reasonable request.
