# Supplementary material for: Oncolytic adenovirus H101 ameliorate the efficacy of anti‐PD‐1 monotherapy in colorectal cancer
Source: Cancer Med. 2022 Jun 28;11(23):4575–87. doi: 10.1002/cam4.4845 (PMC9741988; doi:10.1002/cam4.4845)
Supplement: Supplementary file 1 — Figure S1 Figure S2 [file CAM4-11-4575-s001.pdf]

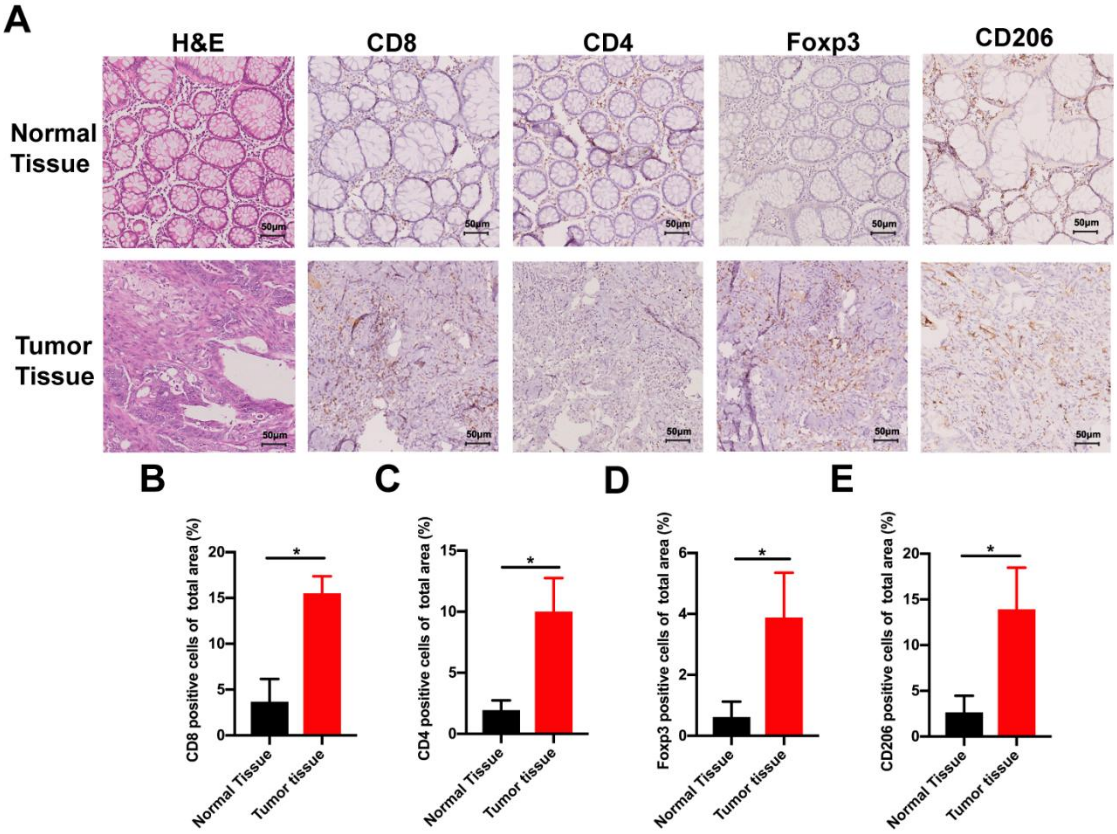

Figure S1. H&E and immunohistochemical staining for CRC tissues and normal tissues. (A) Representative images of tumor samples. Immunohistochemical staining for CD8, CD4, Foxp3, and CD206 in the tumor and normal tissues. (B-E): Quantification of positive cells via imageJ software following CD8 (B), CD4 (C), Foxp3 (D), and CD206 (E) staining. Significance is indicated as * $p < 0.05$.

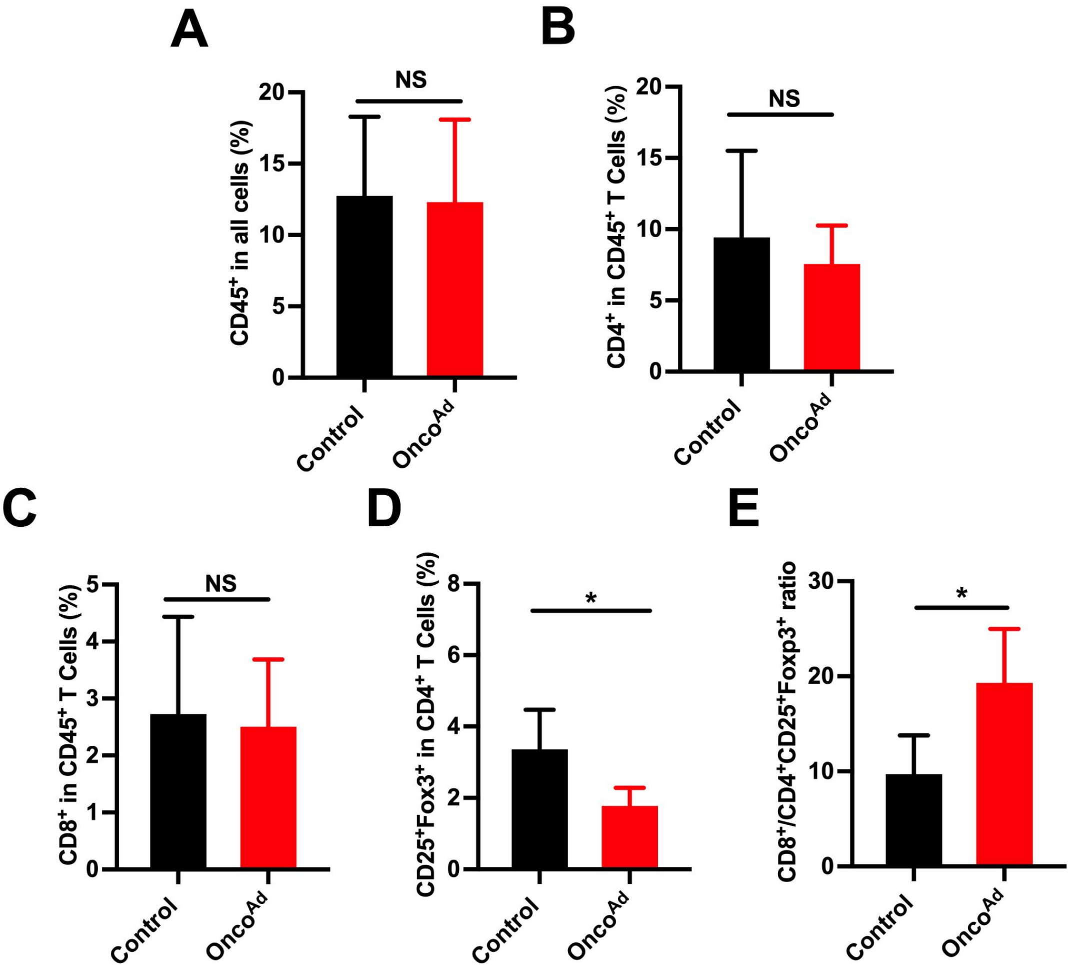

Figure S2. Immune response in the blood after Onco^{Ad} therapy in the CT26 mouse model. (A-E) Percentages of immune cells treated with Onco^{Ad} in the blood. Analysis of the phenotype of CD45^{+} T cells (A), CD4^{+} T cells (B), CD8^{+} T cells (C), and CD25^{+}Foxp3^{+} T cells (D) after Onco^{Ad} therapy. The CD8^{+}/Treg ratio after Onco^{Ad} therapy in the blood (E). Significance is indicated as * $p < 0.05$.
